# Curcumin Improves Diabetic Cardiomyopathy by Inhibiting Pyroptosis through AKT/Nrf2/ARE Pathway

**DOI:** 10.1155/2023/3906043

**Published:** 2023-04-17

**Authors:** Zhang Wei, Kang pinfang, Zhou jing, Yao zhuoya, Qian Shaohuan, Shi Chao

**Affiliations:** ^1^Department of Cardiovascular Medicine of The First Affiliated Hospital of Bengbu Medical College, Bengbu City, Anhui, China 233000; ^2^Department of Cardiac Surgery of The First Affiliated Hospital of Bengbu Medical College, Bengbu City, Anhui, China 233000

## Abstract

This study is aimed at exploring whether curcumin can regulate the AKT pathway, promote the transfer of Nrf2 into the nucleus, and inhibit cell pyroptosis in diabetic cardiomyopathy. Diabetic rats and cardiomyocytes were treated with curcumin to study its effect on myocardial pyroptosis. Whether curcumin can promote the transfer of Nrf2 into the nucleus through AKT pathway regulation was assessed by western blotting and immunofluorescence. The Nrf2 knockout vector and ml385 were used to block the Nrf2 pathway, and the differences between the different groups in the expression of pyroptosis protein, cell activity, and incidence of apoptosis were evaluated to verify the relationship between the effect of curcumin on pyroptosis inhibition and the Nrf2 pathway. Curcumin promoted the transfer of Nrf2 into the nucleus through the AKT pathway and increased the expression of the antioxidant factors HO-1 and GCLC. These effects reduced reactive oxygen species accumulation and mitochondrial damage in diabetic myocardium and inhibited diabetes-induced pyroptosis. However, in cardiomyocytes with a blocked Nrf2 pathway, the ability of curcumin to inhibit pyroptosis was significantly reduced, and the protective effect on the cells was lost. Curcumin can reduce the accumulation of superoxide in the myocardium through AKT/Nrf2/ARE pathway activation and inhibit pyroptosis. It also has a role in diabetic cardiomyopathy treatment. This study provides new directions for evaluating the mechanism of diabetic cardiomyopathy and treating diabetic myocardium.

## 1. Introduction

Diabetic cardiomyopathy is a disease in which myocardial cells are exposed to high glucose concentrations for a long time, while intracellular glycosylation increases. Furthermore, protein and lipid metabolism disorders lead to myocardial cell death, thereby affecting the structure and function of the heart [1, 2]. Metabolic disturbances such as insulin resistance, glucotoxicity, and lipotoxicity adversely affect myocardial energy metabolism, ultimately promoting cardiac hypertrophy and interstitial fibrosis [3]. Patients with diabetes are at higher risk for coronary heart disease, heart failure, and other cardiovascular disorders, and they have significantly higher long-term death rates [4, 5]. Therefore, it is very important to clarify the pathogenesis and treatment of diabetic cardiomyopathy.

The nuclear factor-erythroid 2 p45-related factor 2 (Nrf2) signaling pathway has an important role in regulating cellular homeostasis and protecting cells from oxidative stress [6, 7]. Nrf2 can bind to genes containing ARE sequences in the promoter region to promote the expression of glutathione (GSH), glutamate-cysteine ligase catalyst (GClC), oxygenase-1 (HO-1), glutathione-S-transferases (GST), and other proteins [8, 9]. These proteins are called ARE-like elements. Studies have reported that the PI3K signaling pathway can regulate Nrf2 signaling independently of the kelch-like ECH-associated protein 1 (KEAP1) [10, 11]. PI3K catalyzes the phosphorylation of the phosphatidylinositol 4,5-bisphosphate (PIP2) lipid to generate phosphatidylinositol 3,4,5-triphosphate (PIP3). PIP3 phosphorylates AKT downstream, producing phosphorylated AKT (p-AKT) [12]. p-AKT phosphorylates Nrf2, promotes its dissociation from KEAP1, transfers to the nucleus, combines with ARE sequences, and exerts antioxidant and anti-inflammatory effects [13]. Hyperglycemia promotes the binding of glycosylation products to receptors, activates signaling pathways such as NF-*κ*B, and induces cardiomyocyte production of a large number of inflammatory factors [14, 15]. Long-term insulin resistance leads cells to use fatty acid oxidation for energy production, resulting in an increase in reactive oxygen species (ROS) in the oxidative respiratory chain and impaired mitochondrial function [16, 17]. The accumulation of oxygen free radicals, stimulation of inflammatory factors, mitochondrial dysfunction, and other factors activate NLRP3 and caspase-1, thus causing cells to undergo pyroptosis [18]. Studies have found that activating the Nrf2 pathway can reduce the production of caspase-1 and NLRP3 and reduce the damage to the nervous system caused by magnolol [19]. On the other hand, the ARE-like elements, such as HO-1, GClC, and GST, can participate in the production of the antioxidant factor NADPH and exert antioxidative and anti-inflammatory effects. Therefore, the activation of the ARE-like elements can inhibit pyroptosis to a certain extent.

On the other hand, curcumin is a natural antioxidant that protects cells from inflammatory damage and has therapeutic effects in diseases such as myocardial, kidney, and liver injuries [20–22]. Li et al.'s report found that curcumin can improve mercuric chloride-induced liver injury through the Nrf2/ARE pathway [23]. Zhao et al.'s study found that curcumin inhibited autophagic death caused by HgCl2 through the PI3K/AKT/Nrf2 pathway, thereby improving spleen damage [24]. Therefore, curcumin is a natural agonist of Nrf2, which provides a good pharmacological basis for the treatment of diabetic cardiomyopathy. At present, there is still a lack of studies assessing whether curcumin can inhibit cell pyroptosis through the PIP3/AKT/Nrf2 pathway and play a role in the treatment of diabetic cardiomyopathy. The present study is aimed at experimentally verifying this hypothesis.

## 2. Materials and Methods

### 2.1. Diabetic Rat Model

One-month-old male Sprague-Dawley (SD) rats were purchased from the Experimental Animal Center of Bengbu Medical College. A high-fat diet and intraperitoneal injection of streptozotocin (STZ) were used to establish a diabetic rat model. SD rats were fed a high-fat diet for 1 month before they received the intraperitoneal STZ solution injection (30 mg/kg) [25]. The body weight and blood sugar levels of the rats were monitored regularly. The diabetic rat model was considered successfully established when the fasting blood glucose level was more than 16.7 mmol/L. The normal control group was fed with standard feed without special treatment. According to the purpose of the study, diabetic rats were divided into the following groups: diabetes group, diabetes + curcumin gavage group, diabetes + ml385 injection group, and diabetes + curcumin gavage + ml385 injection group. Eight rats were assigned to each group, and all animals were housed in the animal room of Bengbu Medical College at room temperature (25°C) with 12 h day and night cycles. Rats in each group were housed in separate cages. After grouping, the rats were kept for 8 months. The experimental procedure is shown in the Supplementary Information (available here). The ethical approval of this study was provided by the Animal Ethics Committee of Bengbu Medical College (BBYXY-2021KY03). All rat corpses were sent to the animal room for uniform sterilization after autopsies.

### 2.2. Solutions Preparation

The ml385 solution was prepared to a concentration of 5 mg/mL in DMSO, and the corresponding solution volume was intraperitoneally injected into the rat body according to its weight (20 mg/kg). The curcumin mother solution was prepared at a concentration of 40 mg/mL in DMSO. According to the weight of the rat and considering a daily curcumin dosage of 200 mg/kg, the corresponding curcumin solution volume was mixed with saline to a total volume of 5 mL and injected into the stomach of the rat through a gastric tube. The control groups were injected with 5 mL of normal saline as control.

### 2.3. Separation of Serum and Myocardial Tissue

Rats were sacrificed using phenobarbital anesthetic. The eyeballs were detached for blood collection, and serum was collected after centrifugation to estimate the serum indices. Simultaneously, the heart was dissected, and the apical part was cut and rinsed with phosphate-buffered saline (PBS). After fixation with 4% paraformaldehyde, the myocardial tissue was embedded in paraffin; after dewaxing, hematoxylin and eosin (HE) and Masson's centrifugation stainings were performed.

### 2.4. Extraction of Primary Rat Cardiomyocytes

Suckling rats (1 to 3 days of age) were immersed in alcohol for disinfection, euthanized, and dissected for heart collection. Forceps were used to remove the pericardium and reduce the atrium and blood vessels. Ophthalmic scissors were used to chop the remaining ventricular tissue. A 0.08% pancreatin solution and a 0.2% collagenase II solution were used to dissociate the myocardial tissue. Fibroblasts were removed by differential adherent centrifugation. Cardiomyocytes were then cultured until spontaneous contraction was observed; then, the relevant experiments were performed.

### 2.5. Myocardial Cell Grouping

High-glucose medium containing 30 mmol/L glucose was used to mimic high-glucose-induced cardiomyocyte injury. Myocardial cells were cultured in the medium of conventional 5 mmol/L glucose as the control group. According to the differences in the purpose of the experiment and the treatment process, cells were divided into the following groups: 5.5 mmol/L glucose control group, 30 mmol/L high concentration glucose group, 30 mmol/L high concentration glucose +14 *μ*M/L curcumin group, 30 mmol/L high concentration glucose + Si-Nrf2 transfection group, and 30 mmol/L high concentration glucose +14 *μ*M/L curcumin + Si-Nrf2 transfection group.

### 2.6. Main Experimental Reagents

The One-Step TUNEL Apoptosis Assay Kit (C1086), Cell Counting Kit-8 (CCK-8, C0038), NLRP3 rabbit monoclonal antibody (AF2155), and ROS assay Kit (s0033ss) were purchased from Beyotime Biotechnology (Shanghai, China). Caspase-1 rabbit pAb (A0964), IL-1*β* rabbit pAb (A16288), IL-18 rabbit pAb (A1115), SOD2 rabbit mAb (A19576), and NRF2 rabbit pAb (A0674) were purchased from ABclonal (Wuhan, China). The mitochondrial membrane potential assay kit, JC-1 (M8650), ml385 (IM1020), and nigericin sodium salt (IN1880) were purchased from Solarbio Science (Beijing, China). MitoSOX™ Red mitochondrial superoxide indicator (M6214020) was purchased from Yeasen Biotechnology (Shanghai, China).

### 2.7. Indirect Immunofluorescence Detection

Tissues and cells were fixed with paraformaldehyde. The relevant protein antibodies were incubated overnight at 4°C. After washing off the primary antibody with PBS, cells were incubated in the dark for 2 h with the corresponding secondary antibody. The secondary antibody was washed off with PBS, counterstained with DAPI, and observed under a fluorescent fiber microscope.

### 2.8. CCK-8 Detection of Cell Viability

Primary cardiomyocytes were seeded in 96-well plates, and the relevant experiments were performed after spontaneous beating was observed. The original medium was aspirated, and the experimental reagents from the various groups were added to the cells and incubated for another 24 h. Then, 15 *μ*L of the prepared CCK-8 solution was added to the cells and incubated for 1 h in the incubator. Then, a microplate reader was used to detect the relative cell viability.

### 2.9. TUNEL Staining

Tissue or cell slides were fixed with 4% paraformaldehyde, permeabilized with 0.5% Triton solution for 15 min, and washed with PBS three times. The premixed TUNEL staining solution was added and incubated in the dark for 1 h. Rinsing with PBS was performed three times, and DAPI was added for counterstaining for 5 min.

### 2.10. Primer Design

The sequences of the main PCR primers in this paper are as follows: Nrf2: F-CAGCATGTTACGTGATGAGG, R-GCTCAGAAAAGGCTCCATCC; HO-1: F-TATTCCTGCCCCAATCGCT, R-TTGACCTCCTTCTCGCCCTC; GCLC: F-GATGATGCCAACGAGTCTGA, R-GACAGCGGAATGAGGAAGTC.

### 2.11. Data Analysis

All experiments were repeated at least three times. Data are expressed as the mean ± standard deviation. A *t*-test was used to validate the data of the two groups. ImageJ software (National Institutes of Health, Bethesda, MD) was used to analyze the fluorescence intensity. Differences were considered statistically significant when *P* < 0.05.

## 3. Results

### 3.1. Effects of Different Curcumin Doses on Cardiomyocyte Activity

**Figure 1:**
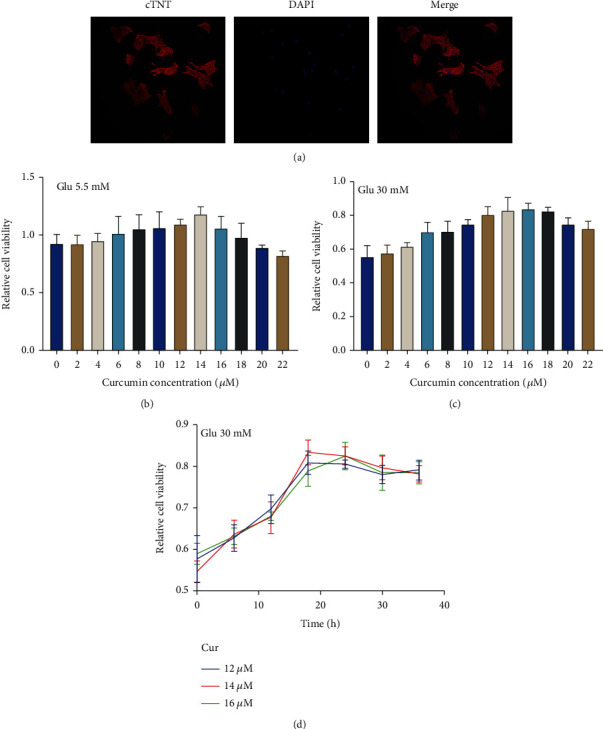
Detection of cardiomyocyte purity and cell viability. Glu: glucose. (a) Troponin was used to identify cardiomyocytes; cardiomyocyte purity was more than 90% (20x). (b) In a 5.5 mM glucose medium, the CCK-8 test was used to measure the impact of various curcumin concentrations on cardiomyocyte activity. (c) In a 30 mM glucose medium, the CCK-8 test was used to measure the impact of various curcumin concentrations on cardiomyocyte activity. (d) Cell viability over time in cardiomyocytes under different curcumin concentrations. Values are displayed as the mean and standard deviation.

Rat primary cardiomyocytes were cultured for 48 h, and spontaneous beating was observed. Cardiomyocytes were fluorescently labeled with troponin, and the purity of the isolated cardiomyocytes was found to be more than 90%, as observed under a microscope (Figure 1(a)). The CCK-8 test was used to identify changes in cardiomyocyte activity under various curcumin and medium concentrations and various coincubation periods to choose the best concentration of curcumin. The CCK-8 test findings demonstrated that the best cardiac viability occurred after coincubation for approximately 18 h with a curcumin concentration of 14 *μ*M/L (Figures 1(b)–1(d)). Therefore, 14 *μ*M/L curcumin was selected as the dosage for the experiment.

### 3.2. Curcumin Inhibits Myocardial Pyroptosis in High-Glucose Medium

**Figure 2:**
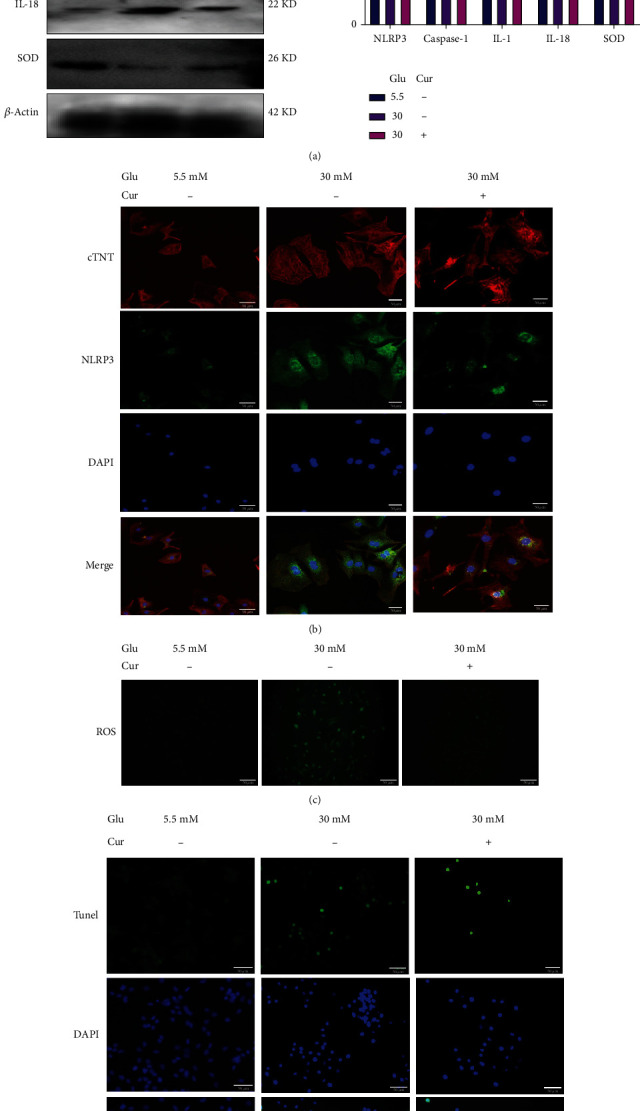
Curcumin-reduced pyroptosis induced by a high-glucose environment. Glu: glucose; Cur: curcumin; the dose of curcumin used in cell experiments is 14 *μ*M. (a) The expression levels of related proteins in the pyroptotic pathway were detected by western blotting. (b) The expression level of NLRP3 in cardiomyocytes was detected by immunofluorescence (40x). (c) The levels of reactive oxygen species (ROS) in cardiomyocytes of each group were detected by ROS fluorescent probing (20x). (d) The apoptosis level of cardiomyocytes in each group was detected by TUNEL fluorescent probing (20x). ^∗∗^*P* < 0.001, high-glucose group vs. normal group and curcumin-treated group. Values are expressed as the mean ± standard deviation.

A culture of cardiomyocytes in a high-glucose medium confirmed the protective effect of curcumin. These findings demonstrated that curcumin dramatically slowed the pyroptosis process and decreased the expression of proteins linked to pyroptosis in 30 mmol/L glucose culture medium (Figures 2(a) and 2(b), *P* < 0.001). At the same time, according to the SOD protein expression and ROS fluorescence analysis, curcumin alleviated the accumulation of superoxide in cardiomyocytes in a high-glucose medium (Figures 2(a) and 2(c), *P* < 0.001). Curcumin attenuated the effects of pyroptosis and ROS scavenging, thereby reducing the incidence of apoptosis in a high-glucose medium (Figure 2(d)).

### 3.3. Curcumin Inhibits Pyroptosis in Diabetic Myocardial Tissue

**Figure 3:**
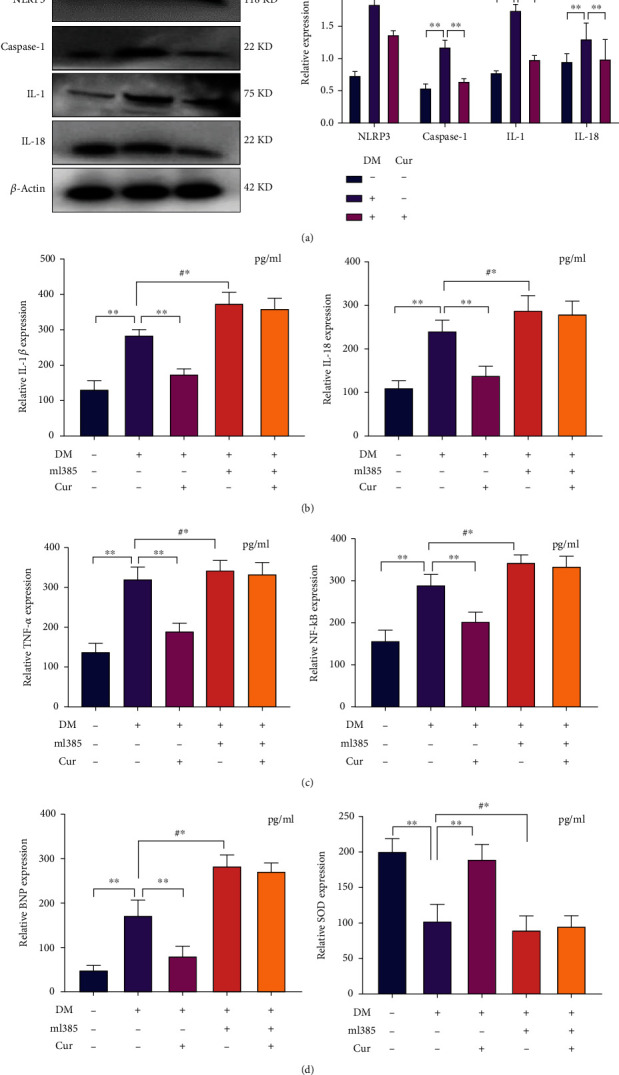
Curcumin inhibits diabetes-induced myocardial pyroptosis. DM: diabetic rat; Cur: curcumin; the dose used in animal experiments was 200 mg/kg/day. ml385: inhibitors of the Nrf2 pathway, the dose used in the experiment was 20 mg/kg. (a) The expression of pyroptosis proteins in rat myocardial tissue was detected by western blotting. (b–d) Expression levels of IL-18, IL-1, NF-*κ*B, TNF-*α*, BNP, and SOD in the serum of the rats in each group were determined using an enzyme-linked immunosorbent assay vs. the control group and curcumin-treated group. #^∗^*P* < 0.001: injected with ml385 vs. noninjected with ml385 (in diabetic rats). Values are expressed as the mean ± standard deviation.

Diabetic rats had significantly increased expression of pyroptosis proteins in myocardial tissue. However, curcumin lowered the expression of proteins linked to pyroptosis in the cardiac tissues of diabetic rats (Figure 3(a), *P* < 0.001). Additionally, curcumin enhanced the level of SOD and further decreased the expression of NF-*κ*B, IL-1, IL-18, TNF-*α*, and BNP in the serum of diabetic rats (Figures 3(b)–3(d), *P* < 0.001). On the other hand, curcumin had a little therapeutic effect on diabetic rats injected with ml385 (Figures 3(b)–3(d), *P* < 0.001). Therefore, we speculated that there was a link between the inhibitory effect of curcumin on pyroptosis and the Nrf2 pathway.

### 3.4. Curcumin Promotes the Translocation of Nrf2 into the Nucleus through the AKT Pathway

**Figure 4:**
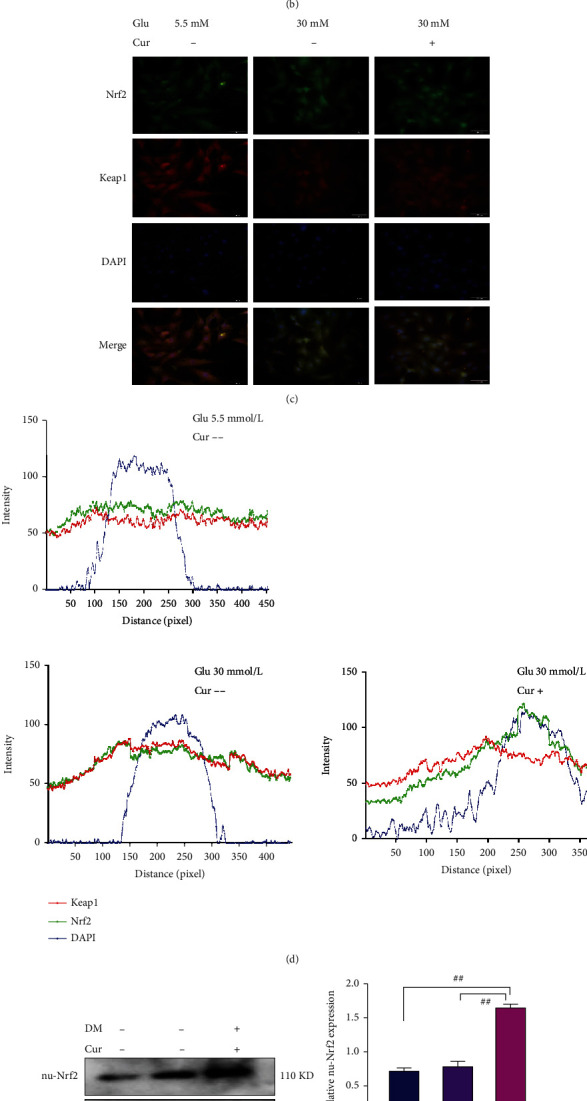
Curcumin promotes the translocation of Nrf2 into the nucleus through the AKT pathway. Glu: glucose; DM: diabetic rat; Cur: curcumin; the dose of curcumin used in cell experiments was 14 *μ*M; the dose used in the rat experiment was 200 mg/kg/day. (a) AKT/Nrf2/ARE pathway protein expression levels in cardiomyocytes were detected using western blotting. (b) The expression of the Nrf2 protein in the nucleus (nu-Nrf2) of cardiomyocytes was detected by western blotting. (c) Expression levels of immunofluorescence-labeled Nrf2 in different parts of cells (20x). (d) The expression sites of the Nrf2, DAPI, and KEAP1 proteins were analyzed for fluorescence colocalization using ImageJ. (e) Western blotting detection of Nrf2 protein expression in the nucleus of rat myocardial tissue. (f) Western blotting was performed to detect the expression levels of proteins in the AKT/Nrf2/ARE pathway in rat myocardial tissue. ^∗∗^*P* < 0.001: high-glucose group vs. normal medium and curcumin treatment group. #*P* < 0.001: curcumin-treated group vs. curcumin-treated group. ^^*P* < 0.001: diabetes group vs. control group and curcumin-treated group. ^^∗^*P* < 0.001: curcumin-fed vs. no curcumin (in diabetic rats). Values are expressed as the mean ± standard deviation.

In in vitro experiments, protein expression in the PI3K/AKT pathway was assessed, and curcumin significantly increased the expression of p-AKT in cardiomyocytes (Figure 4(a), *P* < 0.001). Nuclear Nrf2 protein expression was evaluated by extracting nuclear proteins. In cardiomyocytes, curcumin promoted the translocation of Nrf2 into the nucleus and increased the expression of the downstream antioxidant proteins HO-1 and GCLC (Figures 4(a) and 4(b), *P* < 0.001). Fluorescent antibodies were used to label Nrf2 and KEAP1 in cardiomyocytes, and the fluorescence colocalization analysis further confirmed that curcumin could promote the transfer of Nrf2 into the nucleus (Figures 4(b) and 4(c), *P* < 0.001). Consistent with the results at the cellular level, the expression of p-AKT increased in the myocardial tissue of diabetic rats fed curcumin. p-AKT promoted the translocation of Nrf2 into the nucleus and increased the expressions of HO-1 and GCLC (Figures 4(e) and 4(f), *P* < 0.001). Therefore, this experiment proved that curcumin could promote the transfer of Nrf2 into the nucleus through the AKT pathway.

### 3.5. Suppression of Pyroptosis by Curcumin Was Inhibited by Si-Nrf2 Transfection

**Figure 5:**
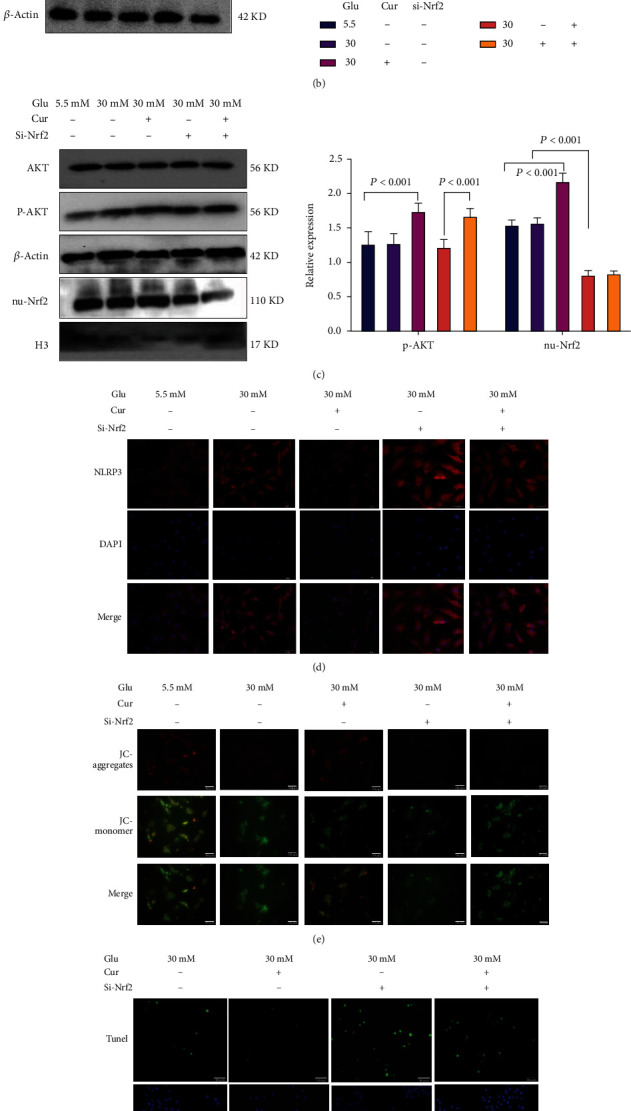
Interfering with the expression of Nrf2 hinders the cardioprotective effect of curcumin. Glu: glucose; Cur: curcumin; the dose of curcumin used in cell experiments is 14 *μ*M. Si-Nrf2: interference virus vector targeting the Nrf2 gene. (a) PCR and western blotting detection of intracellular Nrf2 expression. (b) Western blotting detection of pyroptosis protein expression levels. (c) Western blotting detection of protein expression levels in the AKT/Nrf2 pathway. (d) Immunofluorescence detected the fluorescence intensity of NLRP3 (20x). (e) Changes in the mitochondrial membrane potential levels of cardiomyocytes. The stronger the red fluorescence, the worse the cell viability; the stronger the green fluorescence, the better the cell viability (20x). (f) TUNEL fluorescent probe to detect the incidence of apoptosis (20x). (g) The CCK-8 test detects cardiomyocyte activity. ^∗∗^*P* < 0.001, high-glucose group vs. normal group and curcumin-treated group. #^∗^*P* < 0.001: transfected siRNA vs. untransfected cells (in high-glucose medium). #*P* < 0.05: transfected siRNA vs. untransfected cells (in high-glucose medium). Values are expressed as the mean ± standard deviation.

We further verified the link between curcumin pyroptosis inhibition and the Nrf2 pathway. PCR and western blotting indicated that Si-Nrf2 transfection dramatically decreased the expression of Nrf2 in cardiomyocytes (Figure 5(a), *P* < 0.001). Compared to nontransfected cardiomyocytes, Si-Nrf2-transfected cardiomyocytes were more susceptible to injury induced by high glucose, and the expression of NLRP3, caspase-1, IL-1*β*, and IL-18 proteins was significantly increased (Figures 5(b)–5(d), *P* < 0.001). Moreover, in Si-Nrf2-transfected cardiomyocytes, the degree of mitochondrial damage and level of apoptosis were significantly increased (Figures 5(e) and 5(f)). In cells transfected with Si-Nrf2, curcumin increased the expression of p-AKT but not the translocation of Nrf2 into the nucleus (Figure 5(c), *P* < 0.001), and the activity of cardiomyocytes was not considerably increased (Figure 5(g), *P* < 0.001). In Si-Nrf2-transfected cardiomyocytes, curcumin treatment had no cardioprotective effects.

### 3.6. ml385 Inhibited the Effect of Curcumin on the Treatment of Diabetic Cardiomyopathy

**Figure 6:**
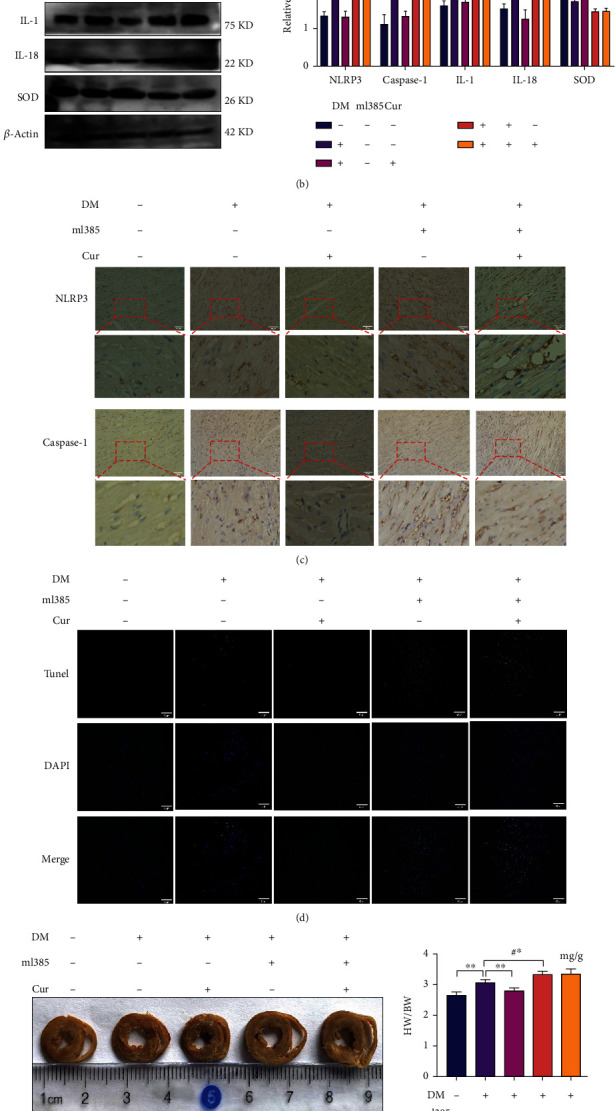
ml385 inhibits flavin protective effects in diabetic myocardial injury. DM: diabetic rat; Cur: curcumin; the dose used in this experiment was 200 mg/kg/day. ml385: inhibitor of the Nrf2 pathway, the dose used in the experiment was 20 mg/kg. (a) PCR and western blotting to verify the inhibitory effect of ml385 on the Nrf2 pathway. (b) Detection of protein expression of the AKT/Nrf2 and pyroptosis pathways in cardiac tissue by western blotting. (c) Myocardial cross-section and the ratio of the heart weight to body weight (HW/BW). (d) Immunohistochemical results of NLRP3 and caspase-1 staining in rat myocardial tissue (20x). (e) TUNEL was used to detect the apoptosis rate of myocardial cells of rats in each group (20x). (f) Hematoxylin and eosin (HE) staining, Masson's staining, and collagen II fluorescence detection of myocardial tissue (20x). ^∗∗^*P* < 0.001: diabetic rats vs. control and curcumin-treated groups. #*P* < 0.05: injected with ml385 vs. not injected (in diabetic rats). #^∗^*P* < 0.001: injected with ml385 vs. not injected (in diabetic rats). Values are expressed as the mean ± standard deviation.

The Nrf2 pathway was suppressed by intraperitoneal injection of the Nrf2 inhibitor ml385 (20 mg/kg). ml385 significantly inhibited the Nrf2 pathway according to the PCR and western blot results (Figure 6(a)). After intraperitoneal injection of ml385 to diabetic rats, the degree of cardiomyocyte pyroptosis caused by diabetes was further aggravated (Figures 6(b) and 6(c)). The effect of curcumin on diabetic cardiomyopathy was significantly inhibited by ml358, and apoptosis was significantly increased in myocardial tissue (Figure 6(d)). Compared with diabetic rats not injected with ml385, ml385 inhibited the effect of flavin on improving myocardial arrangement disorder, edema, and fibrosis. The degree of myocardial fibrosis in the hearts of diabetic rats injected with ml385 was more severe, the hearts were significantly enlarged, and the heart weight-to-body weight ratios were significantly increased (Figures 6(e) and 6(f)). These results confirmed that the pyroptosis inhibition effect of curcumin requires the participation of the Nrf2 pathway.

### 3.7. Curcumin Ameliorates Nigericin-Induced Myocardial Pyroptosis

**Figure 7:**
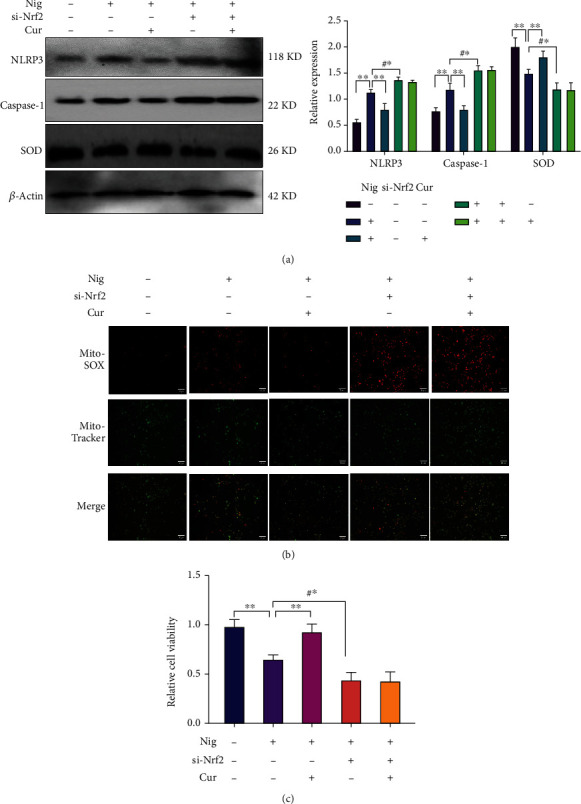
Curcumin ameliorated nigericin-induced pyroptosis. Nig: nigericin; the concentration used in the experiment was 10 *μ*M. Cur: curcumin; the dose of curcumin used in cell experiments is 14 *μ*M. Si-Nrf2: interference virus vector targeting the Nrf2 gene. (a) Western blotting to detect the concentration of pyroptosis-related proteins. (b) MitoTracker detects the relative expression of mitochondrial reactive oxygen species (ROS). The stronger the red, the more obvious the accumulation of ROS in the mitochondria; the mitochondrial tracer is shown in green (20x). (c) The CCK-8 test detected cell activity in each group. ^∗∗^*P* < 0.001: nigericin-added group vs. control group and curcumin-treated group. #^∗^*P* < 0.001: Si-Nrf2 transfected group vs. Si-Nrf2 nontransfected group (in nigericin-supplemented medium). Values are expressed as the mean ± standard deviation.

Nigericin is an inducer of pyroptosis. During this experiment, a concentration of 10 *μ*M was used to induce cardiomyocyte pyroptosis. Curcumin could effectively decrease nigericin-induced pyroptosis and increase the activity of Si-Nrf2 nontransfected cardiomyocytes (Figure 7(a)). In contrast, Si-Nrf2 transfected cardiomyocytes were less resistant to pyroptosis caused by nigericin, and their activity was further reduced (Figures 7(a)–7(c)). In Si-Nrf2 transfected cardiomyocytes, the effect of curcumin on pyroptosis was significantly inhibited. Therefore, our experiment confirmed that curcumin inhibits pyroptosis and requires the participation of the Nrf2 pathway.

## 4. Discussion

Long-term diabetes triggers a variety of inflammatory stimuli in cardiomyocytes, which induce the release of collagen into the myocardium and exacerbate myocardial fibrosis [26]. Advanced diabetic cardiomyopathy leads to structural and functional disturbances in the myocardium, resulting in severe heart failure [27]. Previous studies have found that, in diabetic patients and animal models of diabetic cardiomyopathy, myocardial expression of pyroptotic proteins is significantly increased [28]. In agreement with the previous study, the present study also confirmed that the expression of pyroptotic proteins, such as caspase-3 and IL-1, in the cardiac tissue of diabetic cardiomyopathy was significantly increased. Therefore, controlling pyroptosis is an important target for diabetic cardiomyopathy treatment.

Current studies have found that a variety of protein kinases, among which the research on the PI3K/AKT pathway is the clearest, can participate in the regulation of Nrf2 independently of KEAP1/P53 proteins. PI3K is an important member of the lipid kinase family and cooperates with AKT in a variety of cellular processes, including proliferation, growth, and metabolism [29, 30]. Currently, the PI3K/AKT/Nrf2 pathway is considered an important way for cells to resist oxidative stress. Studies have found that hesperetin can improve the oxidative stress and inflammatory response of liver cells induced by a high-fat diet by activating the PI3K/AKT/Nrf2/ARE pathway [31]. Yang et al.'s research found that metformin alleviated hydrogen peroxide-induced oxidative damage of bone cells and promoted bone cell differentiation through the AKT/Nrf2/HO-1 pathway [32]. In the present study, it was found that activating the AKT/Nrf2/ARE pathway can reduce the progression of pyroptosis in diabetic cardiomyopathy and play a therapeutic role. Therefore, activation of the Nrf2 pathway could serve as an effective target for the treatment of diabetic cardiomyopathy.

The downstream ARE protein group of the Nrf2 pathway has important functions, such as scavenging free radicals, ROS, antioxidation, and anti-inflammation. The Gclc group can participate in the synthesis of GST, and GST can regulate glutathione peroxidase removal of hydrogen peroxide while participating in the removal of oxidation products in the body [33]. Nontoxic concentrations of carbon monoxide generated by HO-1 activity promote mitochondrial biogenesis. HO-1 has a cytoprotective function, which is closely related to its role in regulating inflammatory responses, stress signaling, and mitochondrial protection [34]. As an important scavenger of oxygen free radicals in the body, SOD can reduce the accumulation of ROS in cells and protect the stability of mitochondria. It has important therapeutic value in diseases such as cardiac ischemia-reperfusion injury and heart failure [35]. However, the concentrations of SOD, catalase, and glutathione reductase in the heart tissue are significantly lower than those in other organs, making the heart more susceptible to pyroptosis [36]. In diabetes, factors such as insulin resistance and lipotoxicity can lead to the effects of oxygen free radicals, ROS, and mitochondrial damage, which are precisely the main intrinsic inducers of pyroptosis [37]. Our study confirmed that after curcumin activates the AKT/Nrf2/ARE pathway, it significantly reduces the accumulation of ROS in the myocardium, damage of mitochondria, and degree of myocardial fibrosis. This is closely related to the activation of the Nrf2 pathway, which eliminates the inducers of pyroptosis.

Current research has found that curcumin has a good therapeutic effect on the complications of diabetes. Curcumin can inhibit the NF-*κ*B/p65 pathway in the nervous system of diabetic rats, increasing the activity of superoxide dismutase and reducing the level of inflammatory factors [38]. ALTamimi found that curcumin could inhibit the PKC*β*/p66shc axis and activate FOXO-3a, reversing diabetic nephropathy in rats [39]. The present study found that curcumin can inhibit the progression of pyroptosis in diabetic cardiomyopathy, reduce the degree of myocardial fibrosis, and inhibit the decline of cardiac function by regulating the AKT/Nrf2/ARE pathway. Therefore, our findings support the therapeutic potential of curcumin and provide a new theoretical basis for the treatment of diabetic cardiomyopathy.

## 5. Conclusions

**Figure 8:**
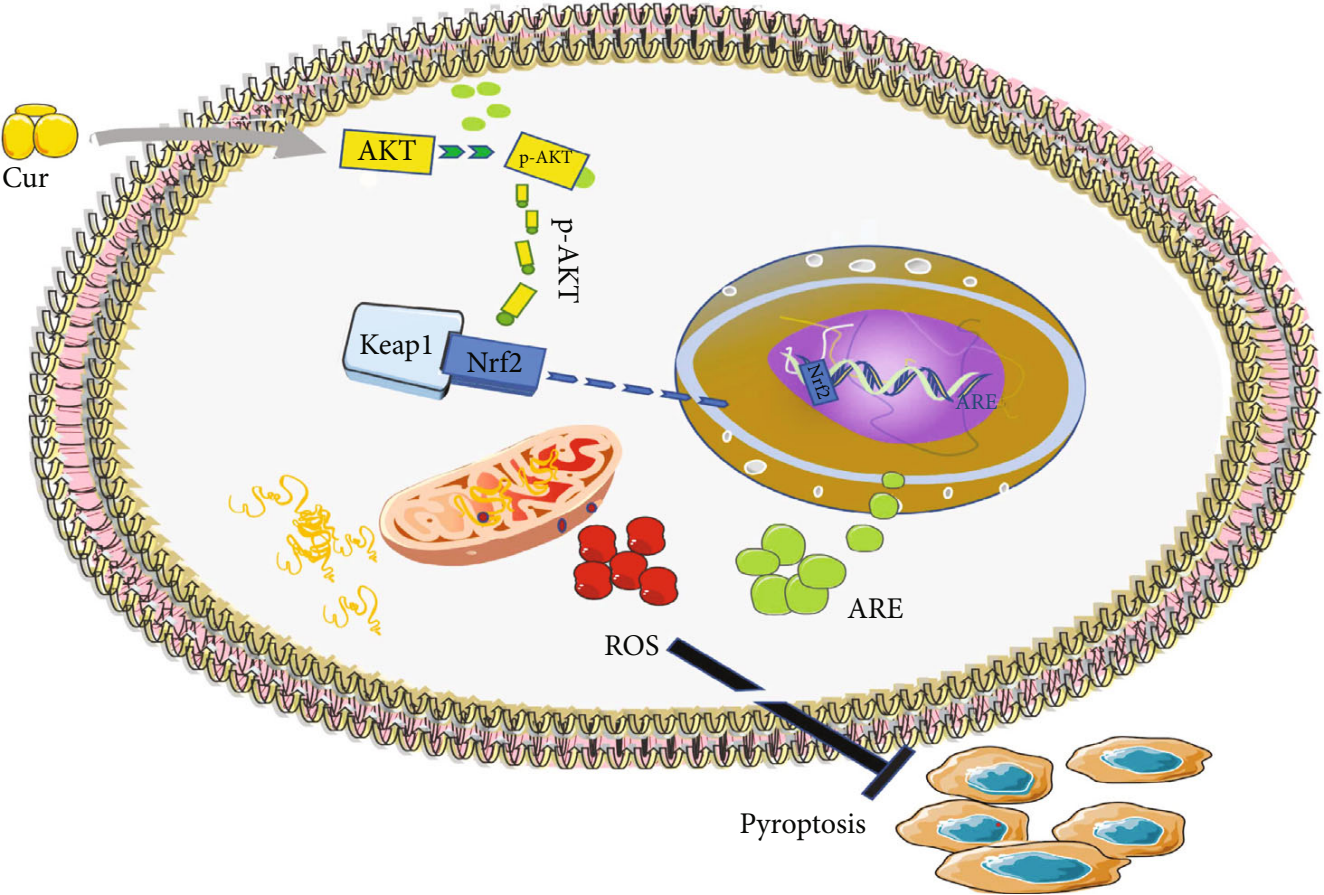
Schematic diagram of curcumin inhibiting pyroptosis through the Nrf2/AKT/ARE pathway.

Diabetic cardiomyopathy is a complication of diabetes. Advanced stages of the disease lead to coronary artery stenosis and decreased cardiac function in patients with cardiovascular disease. This study found that curcumin can promote the transfer of Nrf2 into the nucleus through the AKT pathway and increase the expression of the downstream antioxidant factors HO-1 and GCLC. These effects cleared the accumulation of ROS in diabetic cardiomyocytes, alleviated mitochondrial damage, inhibited the progression of pyroptosis, and had a role in the treatment of diabetic cardiomyopathy. See Figure 8 for the specific mechanism of curcumin action. This study provides a new direction for evaluating the mechanisms of diabetic cardiomyopathy and treating diabetic myocardium.

Diabetes leads to an increase in mitochondrial lipid metabolism and a significant increase in the accumulation of reactive oxygen species, resulting in a pyroptotic response in cells. Curcumin activates AKT phosphorylation, and pAKT promotes the dissociation of Nrf2 from KEAP1 and its translocation into the nucleus. Nrf2 promotes the expression of ARE elements in the nucleus and plays a role in antioxidation and pyroptosis inhibition.

## Data Availability

The raw data supporting the conclusions of this article will be made available by the authors without undue reservation.

## Supplementary Materials

Supplementary Materials Schematic diagram of the establishment of diabetic animal models and grouping of animal experiments.
