# Supplementary figures and images for: Curcumin Improves Diabetic Cardiomyopathy by Inhibiting Pyroptosis through AKT/Nrf2/ARE Pathway
Source: Mediators Inflamm. 2023 Apr 17;2023:3906043. doi: 10.1155/2023/3906043 (PMC10125772; doi:10.1155/2023/3906043)

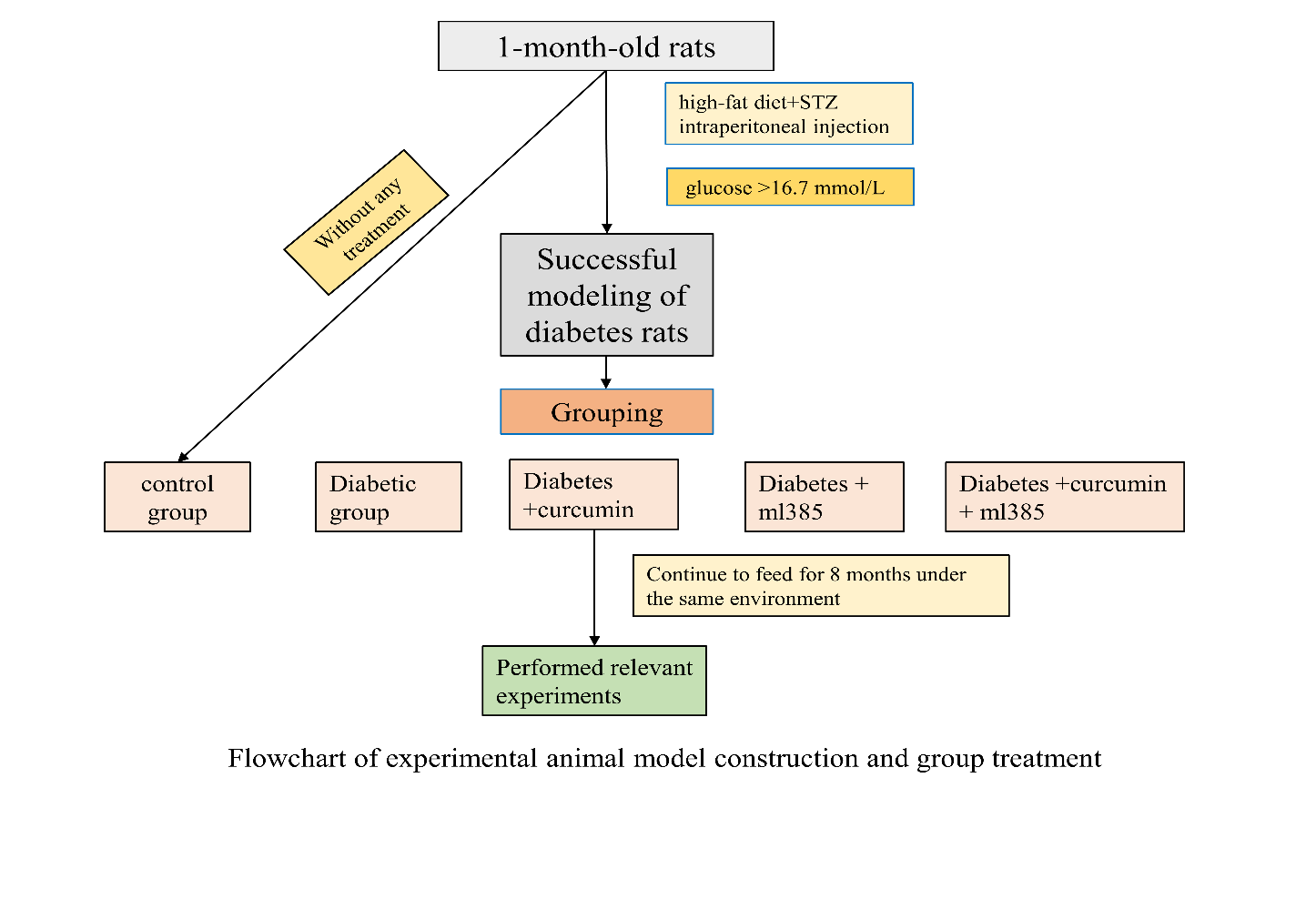

Supplement: Supplementary Materials — Schematic diagram of the establishment of diabetic animal models and grouping of animal experiments. [file 3906043.f1.docx]
